# Magnetization Analysis by Spin-Polarized Scanning Electron Microscopy

**DOI:** 10.1155/2018/2420747

**Published:** 2018-02-04

**Authors:** Teruo Kohashi

**Affiliations:** Research and Development Group, Hitachi, Ltd., 2520 Akanuma, Hatoyama, Saitama 350-0395, Japan

## Abstract

Spin-polarized scanning electron microscopy (spin SEM) is a method for observing magnetic-domain structures by detecting the spin polarization of secondary electrons. It has several unique abilities such as detection of full magnetization orientation and high-spatial-resolution measurement. Several spin-SEM experiments have demonstrated that it is a promising method for studying various types of magnetic materials and devices. This review paper presents several spin-SEM observations to demonstrate the capability and potential of spin SEM.

## 1. Introduction

Direct visualization of magnetization distribution has provided much information about the basic characteristics of magnetic materials, which has promoted the development and improvement of magnetic devices. Several methods for observing magnetization use microscopy. Transmission electron microscopy (TEM), for example, detects the magnetic flux density in a thin film sample using Lorenz microscopy or electron holography. Magnetic force microscopy detects the gradient of a stray magnetic field above the sample surface using probe microscopy. Polarized light microscopy uses the Kerr effect, in which the polarization of the light changes due to the magnetization of the sample. Each method has advantages and drawbacks; therefore, users should use the method that best matches the measurement purpose.

**Figure 1:**
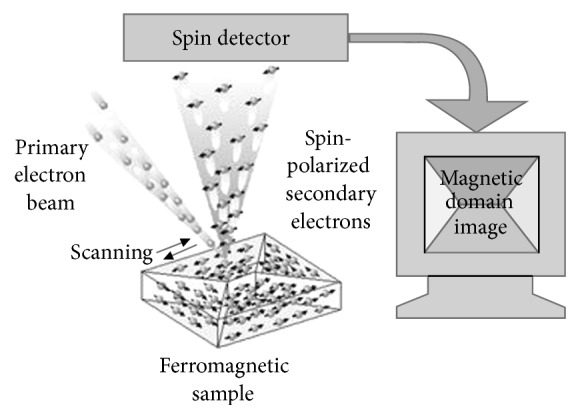
Principle of magnetic-domain imaging by spin SEM.

Spin-polarized scanning electron microscopy (spin SEM) [1–6] is used for detecting the spin polarization of secondary electrons from ferromagnetic samples. The principle is summarized in Figure 1. A probe electron beam is focused on the sample surface, causing secondary electrons to be emitted from the sample. The sample is assumed to be ferromagnetic, and the origin of the magnetization is the spin polarization of the electrons in the sample caused by exchange coupling. The electrons maintain most of this spin polarization when they are emitted as secondary electrons. This spin polarization of secondary electrons is analyzed with a spin polarimeter, into which the secondary electrons are transferred. Thus, the magnetization at the emission point of the secondary electrons can be estimated. Magnetic-domain images are obtained by scanning the sample surface with respect to the probe electron beam.

This magnetic-domain imaging by spin SEM has several excellent characteristics. The spatial resolution is better than 10 nm [7, 8], which is mainly determined by the probe-beam diameter. Not only the magnetic-domain pattern but also the full magnetization orientation can be obtained [9, 10]. Moreover, the magnetization information is not affected by the surface topography in spin SEM; therefore magnetization pattern can be visualized on a fractured surface or patterned device [11]. Spin SEM has thus been used for analyzing various magnetic materials and devices, such as pure metal, oxide, magnetic recording devices, and permanent magnets. In this review paper, several spin-SEM observations are introduced to demonstrate the capability and potential of spin SEM.

## 2. Spin Polarization Detector

The difference between conventional SEM and spin SEM is, in one word, the detector. With conventional SEM, a scintillator or semiconductor is used, and the number or intensity of the secondary electrons is measured. With spin SEM, a Mott polarimeter [12, 13] or a similar polarimeter is used to detect the spin polarization of the secondary electrons. In the Mott polarimeter, fast electrons scatter in the nuclei of heavy atoms such as gold, where electrostatic interaction is the dominant scattering factor. There is, however, interaction between the spin of the electrons and the magnetic field, which is created by the electrons travelling around the nuclei. This interaction is called spin-orbit interaction. Therefore, the scattering direction is affected by electron spin.

**Figure 2:**
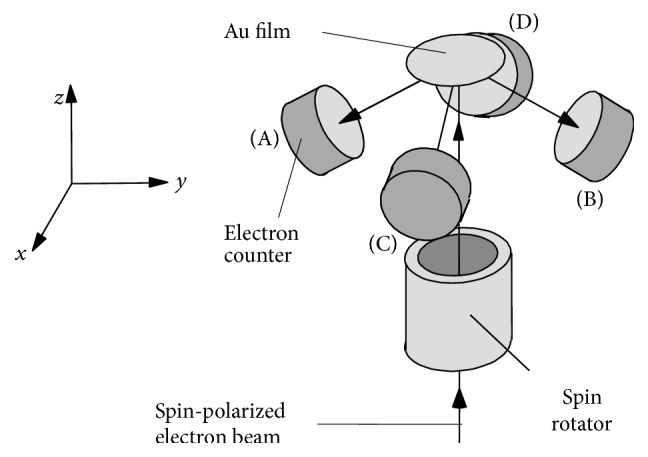
Basic configuration of spin detector (Mott polarimeter).

The basic configuration of our Mott polarimeter is shown in Figure 2. A thin gold film is used as a heavy-atom target, and four electron counters are positioned around the film to detect the backscattered electrons. If the electron beam is spin-polarized in the direction perpendicular to the scattering plane, the number of electrons to be detected by each counter may differ. The two spin polarization components perpendicular to the injected electron beams (*x*- and *y*-components in Figure 2) are calculated using the number of electrons detected by each of the counters. The component parallel to the injected electron beam (*z*-component), however, cannot be detected with the Mott polarimeter. Therefore, we set the spin rotator [10] as shown in Figure 2 and rotated the spin polarization component parallel to the injected electron beam 90° to the detectable direction before the electrons hit the film to enable this component to be detected as well. By switching the spin rotator on and off, all three spin polarization components can be detected.

## 3. Spin-SEM Observations

Examples of magnetic-domain images produced using spin SEM are discussed below.

### 3.1. Co (0001)

**Figure 3:**
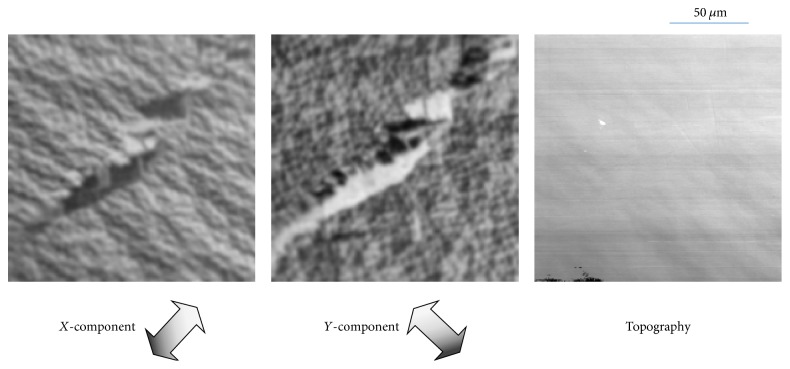
Spin-SEM images of Co (0001) at room temperature. Two in-plane magnetization-component (*X*, *Y*) images and one topography image are shown.

The images in Figure 3 visualize the stress inside a cobalt single crystal. Stress or distortion in the magnetic material changes the magnetic properties, such as anisotropy and permeability; therefore, analysis of these properties is important. In some cases, stress is easier to identify in magnetic-domain images than in topography images.

The sample was Co (0001), which has an easy direction of magnetization along the [0001] crystal axis at room temperature, namely, the direction perpendicular to the observed sample plane. The magnetization, however, inclines in the in-plane direction due to its magnetostatic energy, and closure domains form at the sample surface. In this study we confirmed that the perpendicular component is not very large, so we omitted it.

Two in-plane magnetization-component (*X*, *Y*) images and a topography image are shown in Figure 3. Well-known closure-domain structure [14] can be observed throughout most of the magnetic-domain images; tiny domains (<5 *μ*m long) with various magnetization directions were produced by the hcp structure of the sixfold-symmetry crystal. However, there are large domains (>50 *μ*m long) with intense contrast in the center of the images. The plain structure of the sample is shown in the topography image. The subtle contrast change that is evident in the center of the image corresponds to the area with large structures and intense contrast in the magnetic-domain images. We assume that the large domain structures were caused by local stress in the sample (located in the center of the images). These magnetic-domain images visualize the stressed areas more clearly than the topography image. This analysis of local stress using magnetic-domain observation is important for characterizing magnetic materials.

Analyzing the temperature dependence of magnetic properties is also important because various magnetic devices, such as motors and converters, are often used in high-temperature environments. We installed a sample heating system with a maximum temperature of 500°C in our spin SEM [15] to investigate the temperature dependence of the magnetic properties such as magnetic anisotropy and saturated magnetization.

**Figure 4:**
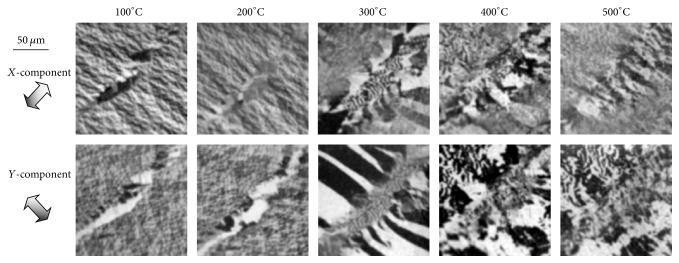
Spin-SEM images of Co (0001) in same area as shown in Figure 3 as temperature was raised from 100 to 500°C. Two in-plane magnetization-component (*X*, *Y*) images are shown for each temperature. Between 200 and 300°C, domain structures of 2-3 um changed into structures larger than 10 um. Between 400 and 500°C, small domain structures of 2-3 um appeared inside large domains.

Figure 4 shows the magnetic-domain structure of Co (0001) in the same area as in Figure 3 from 100 to 500°C. Comparison of the images in Figures 3 and 4 shows that there were domains of 2 to 3 *μ*m and large domains in the area with stress from room temperature to 200°C. Observed changes in the domain structures were small at temperatures up to 200°C but were conspicuous above 300°C. Stripe domains with a width of 10 *μ*m or more appeared in the images taken at 300°C. As the temperature was increased from 400 to 500°C, large domains split into small domains of 2 to 3 *μ*m. The contrast in the *x*- and *y*-components varied for each position of the image, which indicates that the local magnetization direction changed in micrometer scale.

This temperature dependence of the magnetic-domain structure is explained as follows. It is known that the easy axis of the magnetic anisotropy is in the [0001] axis of Co single crystal at room temperature and that it changes into in the (0001) plane above the phase transition (~230°C) [16]. At temperatures below the phase transition, therefore, the magnetization inside the sample is oriented along the [0001] axis, that is, in the direction perpendicular to the sample-surface plane. In this case, the magnetic-domain structure on the sample surface was characterized by small closure domains due to magnetostatic energy. At temperatures above the phase transition, the (0001) plane becomes the easy plane of the magnetization (that is, it is oriented in the sample-surface plane); therefore, large stable domains are both form inside and on the surface of the sample. This phase transition is apparently the origin of the drastic change in magnetic domains that occurred between 200 and 300°C. Moreover, single-crystal Co changed from an hcp structure to an fcc one at around 450°C [17]; therefore, it is assumed that this structure change was related to the changes in the domain structure from 400 to 500°C.

Using spin SEM in this way to observe the magnetic domains on the Co (0001) surface at various temperatures helps in investigating the magnetic properties associated with reported phase transitions.

### 3.2. HDD Medium

**Figure 5:**
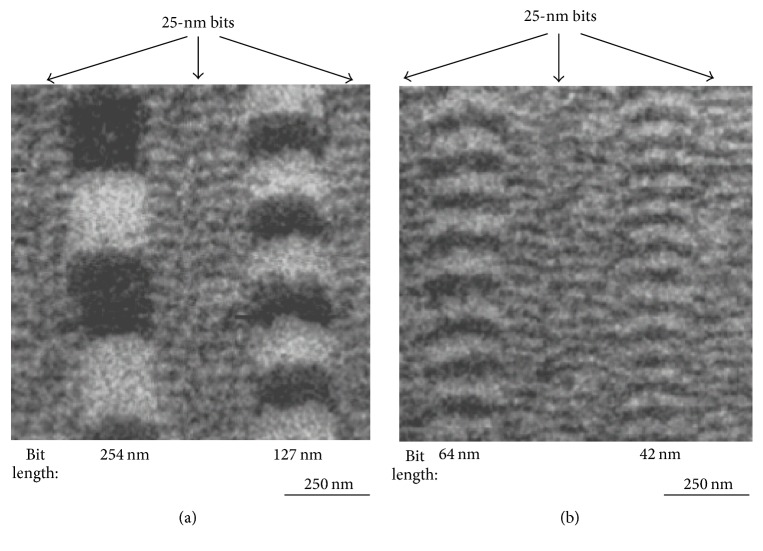
Magnetic-domain images of recorded bits of various lengths: (a) 254 and 127 nm, (b) 64 and 42 nm, and (both in (a) and (b)) 25 nm. A short bit length of 25 nm recorded as background can be clearly observed between the main signals. Reproduced from [7] with permission.

Magnetization of a very small area plays an important role in some cases, such as those involving defects, dislocations, or grain boundaries. The spatial resolution is thus important for microscopic magnetization analysis. The lateral resolution of spin SEM is better than 10 nm, and Figure 5 shows examples of high-resolution magnetic-domain observations [7]. The sample was a perpendicular high-density magnetic recording medium (CoCrPt). Main-signal bits with lengths of 254, 127, 64, and 42 nm were recorded over background bits with a length of 25 nm. Figures 5(a) and 5(b) show the magnetic-domain images obtained using spin SEM; the dark-and-bright contrasts show the magnetization component perpendicular to the sample-surface direction. Tracks run vertically in the image, and each black and white area in the main tracks denotes one recorded bit. We can see that the main signals were clearly recorded. Moreover, tiny bits with a length of 25 nm that were recorded as background can also be identified between the main tracks.

**Figure 6:**
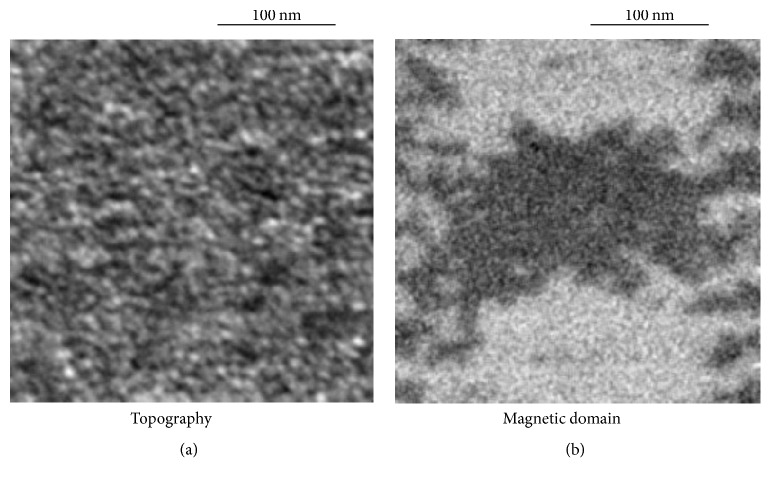
Spin-SEM images of recorded medium: topography and magnetic-domain images of perpendicular magnetization component. Reproduced from [7] with permission.

Figure 6 shows high-magnification topography (a) and magnetic-domain (b) images of the 127-nm bits in the main tracks, which were obtained at the same time in the same field of view [7]. In Figure 6(a), fine structures of 10 nm or less are visualized, which were later determined to be the grain structures of the medium by TEM measurement. On the other hand, the shapes of the 127-nm recorded bits are clearly visible in Figure 6(b), where very fine structures were visualized around the bit boundaries and track edges, and the sizes of the small structures are similar to those of the grain size. Two advantages of spin SEM are demonstrated in these images. The first is that magnetization information is independent of the sample-surface topography. The second is the high-spatial resolution better than 10 nm, which enables investigation of the magnetization of each small grain by comparing the topography and magnetic-domain images. These advantages are applicable to other research areas such as permanent magnets, steel, and metallurgy.

### 3.3. NdFeB-Sintered Magnet

The probing depth of spin SEM is quite short, for example, 1 nm [8], which enables the determination of the magnetization of a very thin sample. We introduce the magnetization measurement of the grain-boundary phase in an NdFeB-sintered magnet by taking spin-SEM images of a fractured surface. Coercivity is an important property of a permanent magnet, and the magnetization of the grain-boundary phase of such magnets has a significant effect on coercivity; therefore, it has been attracting attention. It is not easy, however, to determine the magnetization of the grain-boundary phase of this type of magnet separately from that of the main phase because the thickness of the boundary phase is only several nm while the grain size is several micrometers. The magnetization of the grain-boundary phase is expected to be smaller than that inside the grains (the magnetization of Nd_2_Fe_14_B is more than 1 T at room temperature). Additionally, we should be careful to prevent deterioration in the magnetization during measurement because the grain-boundary phase of this type of magnet is easily oxidized. In these situations, spin-SEM measurement was carried out by taking advantage of its short probing depth. We fractured the sample in an ultrahigh-vacuum chamber to expose the grain-boundary phase on the fractured surface and measured its magnetization before the fracture surface was oxidized [11]. 

**Figure 7:**
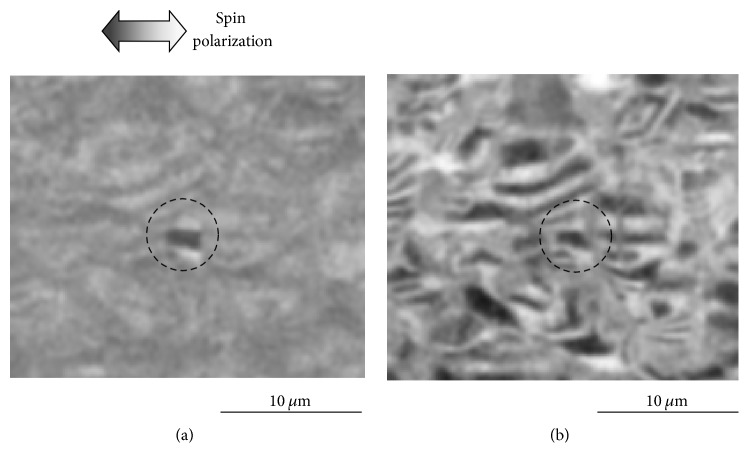
Spin-SEM images of fractured surface of NdFeB-sintered magnet (a) before and (b) after ion milling. Dashed circles show grain fractured on inside. Reproduced from [11] with permission.

In this experiment, the sample was assumed to fracture either at the boundary phase between grains or inside grains. Therefore, various contrasts due to the spin polarization of the secondary electrons were observed for each area in the spin-SEM images. An example of a spin-SEM image on the fractured surface of the magnet is shown in Figure 7(a). The dark-and-bright contrasts in this image show the magnetization component in the grain-oriented direction (horizontal direction in the image). As expected, we can see that there is an area with rather strong contrast in the center of the image, while other areas have weaker contrast. We studied the composition at each point on the fractured surface by using Auger spectroscopy and found that the areas with weak contrast were covered with the grain-boundary phase and that the areas with strong contrast were fractured surfaces inside the grains where the main phases (Nd_2_Fe_14_B) were exposed. The sample surface was milled substantially with Ar ions after the image shown in Figure 7(a) was obtained, and another spin-SEM image of the same area was obtained (Figure 7(b)). In this image, almost the entire area shows strong contrast because the milling removed the thin grain-boundary phases, and magnetization of the main phase was visualized. The measurement details are explained in a previous report [11] of a study in which we determined the magnetization of the grain-boundary phase of the magnet quantitatively by spin-SEM measurement and by gradually milling the fractured surface by using Ar-ion sputtering.

## 4. Conclusion

Spin SEM is a method of observing a magnetic domain with unique characteristics. It has been applied not only for fundamental investigations but also for the development of various magnetic devices. Many interesting studies have been reported, and the method's effectiveness is still being improved such as by increasing its spatial resolution (it is now 3 nm [8]) and by adding various functions. For further such improvement, the efficiency of the spin detector should be improved. The spin detector has not been changed drastically in the past 30 years, and a more efficient spin detector would reduce the amount of time needed for data acquisition, increase the spatial resolution, and improve the image quality. In addition, in situ observations with changes in temperature or magnetic field, such as those described above, will also be important for spin-SEM observation in the future.
